# Lung ultrasound supports clinical evaluation of feeding competence development in preterm neonates

**DOI:** 10.3389/fped.2023.1222473

**Published:** 2023-09-20

**Authors:** Tiziana Controzzi, Francesca Chesi, Rosa Teresa Scaramuzzo, Matteo Giampietri, Riccardo Morganti, Simona Fiori, Elena Moretti, Luna Gargani, Luca Filippi

**Affiliations:** ^1^ U.O. Neonatologia, Dipartimento Materno-Infantile, Azienda Ospedaliero Universitaria Pisana, Pisa, Italy; ^2^ Sezione di Statistica, Azienda Ospedaliero Universitaria Pisana, Pisa, Italy; ^3^ Dipartimento di Medicina Clinica e Sperimentale, Università di Pisa, Pisa, Italy; ^4^ IRCCS Fondazione Stella Maris, Calambrone, Italy; ^5^ Department of Surgical, Medical and Molecular Pathology and Critical Care Medicine, University of Pisa, Pisa, Italy

**Keywords:** preterm infants, lung ultrasound, feeding, deglutitory apnea, inhalation, gastroesophageal reflux, blines

## Abstract

**Introduction:**

The achievement of alimentary competencies is a milestone in the development of preterm neonates. Ten percent of neonates <37 weeks of gestational age and 25% of those VLBW experience swallowing disorders, with an increased risk of problems in the early phase of life (failure to thrive, growth retardation, inhalation, and consequent risk of pulmonary infection) and later in life due to delayed development of oromotor skills.

The main diagnostic tools for swallowing disorders are endoscopic (fiber-optic endoscopic examination of swallowing, FEES) or radiographic (videofluoroscopic swallowing study, VFSS) exams. Given the invasiveness of these methods and the bias due to rheologic differences between bolus and contrast medium, FEES and VFSS are poorly reproducible. Moreover, neither of the technique is capable of detecting post-meal inhalations, especially microinhalations or those consequent to a whole meal rather than to a single swallowing.

Lung ultrasound (LUS) is a widespread, repeatable, safe, fast point-of-care tool and we reported previous encouraging results in detecting silent and overt inhalation related to the meal in children with dysphagia/gastroesophageal reflux disease (GERD) risk factors.

**Methods:**

We report a pilot study, that investigated LUS approach (performing imaging before and after meals) to assess feeding competence development in a cohort of *n*. 19 newborns <32 weeks of age.

**Results:**

Meal monitoring by LUS did not show any significant difference in scoring before/after eating. The achievement of full enteral feeding correlates with GA at birth (*p* < 0.001) but not with LUS scoring. The introduction of the first meal by bottle correlates both with gestational age (*p* < 0.001) and ultrasound scores (*p* = 0.004). LUS score at 7 days of life resulted predictive for length of invasive/non-invasive respiratory support (*p* = 0.002) and length of oxygen supply (*p* = 0.001), while LUS score at 48 h of life did not (*p* n.s.).

**Discussion:**

Our study suggests that the development of oral feeding skills is not strictly dependent on gestational age. Moreover, our research suggests the predominant role of LUS in predicting the time of readiness to oral feeding, as the LUS score can be a marker of respiratory and lung wellness, and consequently a predictor of neonate stability during deglutitory apnea.

## 1. Introduction

The achievement of alimentary competencies is a milestone in the development of preterm neonates. Deglutition disorders can be transient and considered as physiologic during normal maturation. However, preterm infants are a population at risk for real swallowing problems. Indeed, feeding difficulties in premature infants born less than 37 weeks of gestational age (GA) is about 10.5%, which increases to about 24.5% among those born at less than 1,500 grams. This percentage increases even more in case of neurodevelopmental delay, so that feeding difficulties are prevalent in about 80% of preterm infants associated with neurological impairment and are a major reason for chronic clinic visits (1).

When oral feeding milestones are impaired, it is often interpreted as deglutition disorders with varying specific entities, such as feeding difficulties, swallowing disorders, aerodigestive illness, and aspiration syndromes. Related symptoms are heterogeneous, with an augmented risk of problems in the early phase of life (failure to thrive, growth retardation, inhalation, and consequent risk of pulmonary infection) and later in life due to delayed development of oromotor skills (1). Fortunately, feeding difficulties mostly disappear within four years (2) but the consequences they have caused can still determine a deterioration in the quality of life and cause significant morbidity with an independent course. Lack or delay in diagnosis and taking care of the problem leads to an increased risk of persistent swallowing abnormalities in early infancy: in fact, it is estimated that more than 40% of pediatric patients with swallowing disorders report prematurity in their clinical history (3).

During hospitalization in the Neonatal Intensive Care Unit (NICU) and after discharge, strategies to minimize aerodigestive disorders include supporting non-nutritive sucking, developing infant-directed feeding protocols, sensory oromotor stimulation, and early introduction of oral feeds (4). In any case, they are often managed empirically and many controversies still remain in Literature.

Challenges even start from the diagnostic approach. Nowadays, the main diagnostic tools for swallowing disorders are endoscopic (fiber-optic endoscopic examination of swallowing, FEES) or radiographic (video-fluoroscopic swallowing study, VFSS) exams. Given the invasiveness of the methods and the bias due to rheologic differences between bolus and contrast medium, FEES and VFSS are poorly reproducible. In preterm infants, aspects related to maturation would require repeating these exams several times, but evidently this is unreliable. Moreover, neither of the technique is capable of detecting post-meal inhalations.

Lung ultrasound (LUS) is a widespread, repeatable, safe, fast point-of-care tool (5) that is increasingly applied in the NICU facilitating prompt diagnosis and intervention and providing real-time information on pulmonary conditions such as respiratory distress syndrome, transient tachypnea of the newborn, meconium aspiration syndrome, pneumonia, pneumothorax, and pleural effusion. Over the last few years, LUS has also been used as a semi quantitative method since scores have been proposed to monitor the progress of neonatal lung diseases (6) and to decide whether to perform a specific treatment as well as surfactant therapy, respiratory support and drugs against the progress of bronchopulmonary dysplasia (7).

Our research group reported previous encouraging results in detecting silent and overt inhalation related to the meal in children with cerebral palsy or other encephalopathies leading to dysphagia/gastroesophageal reflux disease (GERD) (8) and designed a randomized controlled trial in order to investigate LUS-monitored meals evaluation and feeding management in infants aged 0–6 years with neurodevelopmental delay, the LUNCH Study (ClinicalTrials.gov Identifier: NCT04253951) (9).

The present study aims to propose the same approach to a different at-risk population in a NICU. Our goal was to preliminarily assess the usefulness of LUS as an integrative approach to the management of feeding skills development in at risk populations such as preterm babies, where silent aspiration is frequent (10). As we have already reported above, neonatologists must carefully monitor the progressive development of feeding skills in preterm infants as this impact nutritional status and contribute to chronic pain, poor tone, poor self-pacing, increased respiratory rate, and apnea (3, 11). Indeed, the neuronal network controlling the ability to suck is formed and functional by the 28th week of gestation, but continues to evolve afterwards. Moreover, sucking requires coordinated suck-swallow-breathe actions and this requires an enormously complex sensorimotor process. Around the 30th week of gestation there is the appearance of coordination between sucking and swallowing, initiating a maturational process which becomes complete around 40 weeks of gestational age (12, 13). During fetal life, a gender difference is observed, with an earlier onset of oro-motor capacity in the female sex (larynx-pharyngeal motility and lingual movements), a difference that is no longer detectable in the third trimester of gestation (14). Finally, newborn feeding behavior showed an association with motor developmental outcomes at 4–5 years, so the identification of biomarkers related to secondary complication of feeding disfunction might contribute to a prompt identification of at risk subjects to start early intervention strategies (15, 16). Clinical assessments allow evaluators to score the oral-motor pattern, but they remain ultimately subjective. Thus, objective measures to identify newborns at a real risk of swallowing disorders are needed, in order to tailor feeding strategies, specific therapies (including pacifiers, cheek/chin support, tactile, oral kinesthetic, auditory, vestibular, and/or visual sensorimotor inputs) and give an anticipatory guidance to parents.

Our aim was to contribute to this major objective. Indeed, we intended to explore LUS in the evaluation of feeding disorders in preterm newborns. Our working hypothesis is based on data showing that these patients are at risk of apparent and silent inhalations (17). Therefore, we performed ultrasound monitoring of meals by preprandial and postprandial scans searching for the possible onset of meal-related ultrasound alterations, suggestive of inhalation. In particular, our primary objective was the identification of even silent inhalation episodes by LUS, while our secondary aim was to investigate LUS predictivity with respect to oral feeding capacity, length of hospitalization, pulmonary outcomes, and growth.

## 2. Materials and methods

All inborn and outborn neonates at the NICU of Pisa University Hospital were enrolled, from 1 June to 30 August 2020.

Inclusion criteria were:

i. GA <32 weeks
ii. Written informed consent signed by both parents

Exclusion criteria were:

i. Major malformations
ii. Failure in reaching full enteral feeding
iii. Denied consent by parents

Enrolled newborns were investigated by LUS:

- At 48 h of life (*T*_0_)
- At 7 ± 1 days of life (*T*_1_)
- When eating 20–30 cc/kg/day of enteral nutrition (maternal or donor milk, or formula), i.e., when trophic enteral feeding becomes nutritive enteral feeding (*T*_2_)
- When eating 70–90 cc/kg/day of enteral nutrition (*T*_3_)
- When reaching full enteral feeding (*T*_4_), whether by mouth or tube
- At the beginning of their oral feeding, i.e.,1st meal by bottle (*T*_5_)
- At the achievement of total oral feeding (*T*_6_)
- In the 7 days preceding discharge (*T*_7_)

Every evaluation (except for *T*_0_ and *T*_7_) included LUS before the administration of enteral feeding and another on 15–30 min after meal. Despite the benefits and widespread use of enteral tube feeding, some patients experience complications including aspiration pneumonia due to gastric retention and regurgitation, as reported in literature (18). Therefore, LUS monitoring can be a useful tool to monitor silent or apparent aspiration when infants are fed both by mouth and by tube.

We used two ultrasound scanner: (i) Vividiq (GE, General Electric) with a GE 12l linear probe, and (ii) GE Voluson 8 ultrasound with a GE 11l linear probe. Both tools have a dedicated preset for lung ultrasound. LUS was performed by a neonatologist expert and trained in ultrasound scan (always the same operator, in order to avoid bias due to variability).

LUS was performed in the prone and supine position, obtaining three scans per lung (anterior, lateral, and posterior part of hemithorax by using parasternal line, anterior and posterior axillary lines, and spine as landmarks). Each scan was scored as reported in Bouhemad, 2015 (19) resulting in a total score between 0 and 18. The score is made by the presence and semi-quantification of B-lines, and consolidation.

In a normal lung is it possible to identify a bright line that moves during the patient's breath: that's the pleural line. Under the pleural line are visible multiple horizontal lines without motion, called A-lines. The space between the two of them is regular, as they are the expression of the reverberation of pleural line in a well-aerated lung. So, A-lines are not anatomical findings but an artifact due to the presence of air under the pleural line as in the normal lung.

If the fluid content of the interstitial space increases, brilliant vertical lines starting from the pleural line and extending to the edge of the screen erasing A-lines can be seen. They are called B-lines and can be single, multiple, or coalescent. The more numbered the B-lines are, the more the fluid content in the interstitial space is.

Lastly, consolidations are the expression of a de-aeration of the lung and can be identified by the presence of a “tissue-like” signal underneath the pleura.

**Figure 1:**
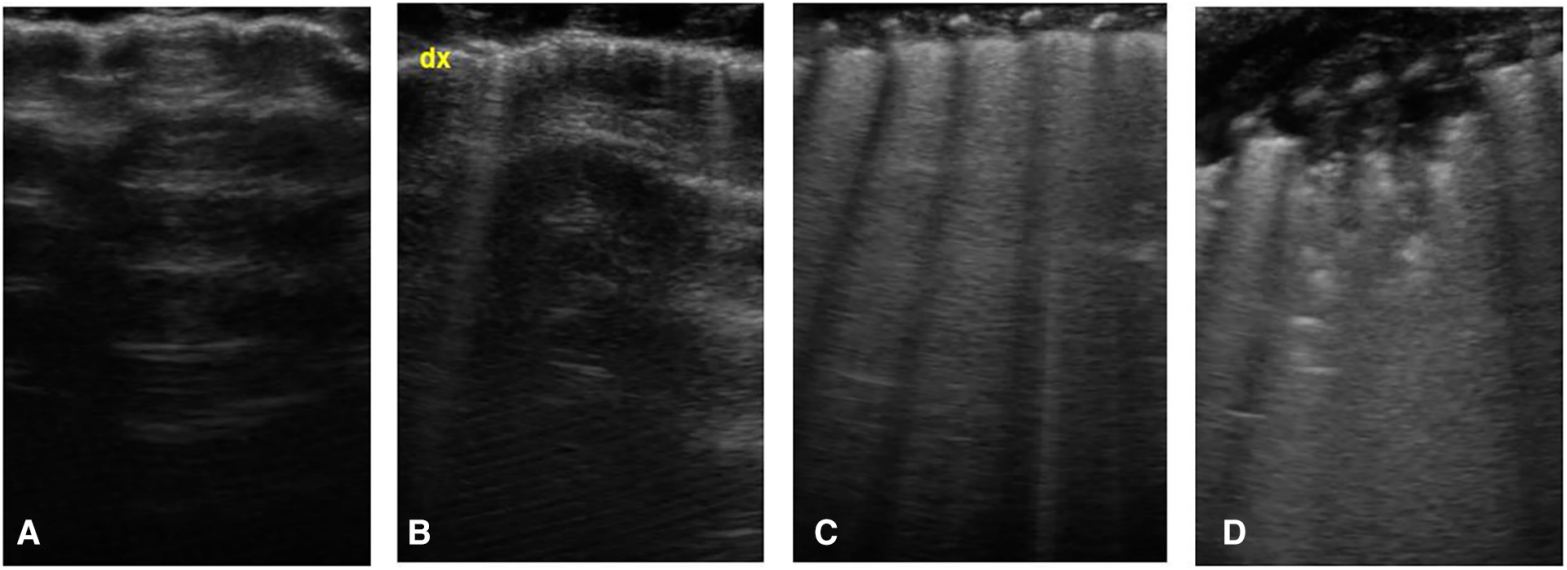
Lung ultrasound score, images obtained by longitudinal scan with a linear probe: (**A**) presence of only A-lines under the pleural line (normal pattern, score 0); (**B**) isolated vertical bright line, starting from pleural line and erasing the underlying A-lines, called B-line (score 1); (**C**) coalescent B-lines (score 2); (**D**) presence of consolidation (score 3).

Based on these findings, each area of the scan can be scored according to this scheme (Figure 1):

- Score 0: A-lines pattern (normal pattern)
- Score 1: isolated B-lines
- Score 2: coalescent B-lines
- Score 3: consolidation

The total score ranges from 0 (normal pattern in each zone) to 18 (presence of consolidation in each zone).

In this study, due to the frequent finding of B-lines in preterm babies and in order to underline the difference between isolated B-lines and multiple B-lines, a modified score was used, as the following:

- Score 0: A-lines pattern (normal pattern)
- Score 1: isolated B-lines (<5 B-lines)
- Score 2: multiple B-lines (>5 B-lines) and/or coalescent B-lines
- Score 3: consolidation

We collected a clinical data set about nutrition:

- Meal administration method (nasogastric tube or bottle)
- Type of milk (maternal or donor milk, fortified human milk, formula)
- Number of meals/day on the day of the exam
- Milk volume per single meal
- Total amount of milk administered throughout the day (ml/kg/day)
- Any symptom/adverse event during the meal (cough, reduction of SpO2 ≥10%, bradycardia with reduction of heart rate ≥30 beats/min)
- Need for respiratory support or oxygen administration (FiO_2_%)
- Drug therapy on course

Meals were administered by expert nurses.

Moreover, for all patients we collected general clinical data:

- GA at birth
- Weight at birth, 14, 28 and 42 days of life and at hospital discharge (evaluated based on INTERGROWTH-21st standards)
- Need of any respiratory support (mechanical ventilation, non-invasive respiratory support, surfactant administration)
- Major morbidities along hospitalization (anemia requiring transfusions, early or late-onset sepsis, patent ductus arteriosus, intraventricular hemorrhage, retinopathy of prematurity, bronchopulmonary dysplasia)

In case of clinical suspicion for inhalation, patients underwent FEES and/or VFSS, according to current gold standard diagnostic flow chart.

Statistical analysis was performed by SPSS v.26 software. Categorical data were described as absolute frequency and percentage, while continuous data as mean and standard deviations. To identify predictive factors of quantitative clinical outcomes, a univariate analysis (Pearson's correlation) was first performed and then a multivariate one (multiple linear regression). The statistical significance (*p*) was set at 0.05.

The study was approved by Tuscany Regional Pediatric Ethical Committee (*n*. 107/2019).

## 3. Results

Overall 19 preterm newborns were enrolled. Among this cohort, 3 neonates were excluded because of major malformations or genetic syndromes. Throughout the study, 1 neonate more dropped out because transferred to another hospital due to a surgical emergency, before having achieved full enteral feeding. General clinical features are shown in Table 1.

**Table 1:** General clinical features of study population.

M/F	10/6	Outcome for weight (Z score)	−0.99 (−2.76–0.08)	Surfactant	13/16
GA (days)	205.6 (179–223)	Sepsis	4/16	Mechanical ventilation	6/16
BW (grams)	1,284.5 (750–1,792)	Blood transfusions	6/16	Respiratory support (days)	28.94 (4–86)
percentile BW	55.93 (18–94)	IVH	0/16	Oxygen dependance (days)	26.06 (2–86)
Time to regain BW (days)	10.875 (6–18)	PDA	4/16	Start enteral feeding (days)	2.83 (1–6)
Weight at 14 days of life (percentile)	31.31 (2.36–80.1)	ROP	3/16	Full enteral feeding (days)	19.44 (7–47)
Weight at 28 days of life (percentile)	21.36 (0.43–66.5)	BPD	6/16	1st meal by bottle (days)	28.93 (12–68)
Outcome for weight (percentile)	24.85 (0.09–53.29)	Steroids and/or diuretics	6/16	Total oral nutrition (days)	37.36 (18–79)

GA, gestational age; BW, birth weight; IVH, intraventricular hemorrage; PDA, patent ductus arteriosus; ROP, retinopathy of prematurity; BPD, bronchopulmonary dysplasia; M/F, male/female.

*T*_0_ LUS score was 10.2 ± 3.2 (medium ± standard deviation; 10.8 ± 3.5 in <30 weeks group, 9.8 ± 3.1 in >30 weeks group), and *T*_1_ score was 10 ± 3.68 (12.2 ± 3.3 in <30 weeks group, 8.5 ± 3.2 in >30 weeks group) (*p* = n.s.).

As forwards LUS monitoring of the meal, no difference was recorded in preprandial vs. postprandial scores (*T*_4_ LUS preprandial score 8.61 ± 3.05–10.1 ± 3.3 in <30 weeks group, 7.6 ± 2.6 in >30 weeks group, *T*_5_ LUS preprandial score 7.47 ± 3.60–9.5 ± 4.2 in <30 weeks group, 6.4 ± 2.5 in >30 weeks group, *T*_6_ LUS preprandial score 5.38 ± 2.53–5.2 ± 2.6 in <30 weeks group, 5.4 ± 2.5 in >30 weeks group).

Episodically, when they were already at full enteral feeding, 2 patients experienced regurgitation over a long time after a meal, with associated symptoms (desaturation and bradycardia, needing for mask ventilation to stabilize vital signs). LUS executed immediately after this episode showed abnormalities in both cases. In one neonate we showed a subcentimeter consolidation in the right paravertebral zone and a similar one in the right posterior apical zone. After 6 h, LUS showed a slight attenuation of the interstitial hyperechogenicity but persistent consolidations.

In the other neonate, LUS after regurgitation showed two persistent symmetrical dysventilated areas in the subscapularis zone, along the posterior axillary line.

Both neonates underwent FESS, that resulted normal, with no evidence of aspiration.

Performing a step-wise multiple linear regression analysis, we evaluated the time for achieving the main nutrition milestones (i.e., full enteral feeding, start of bottle feeding, total oral feeding) related GA at birth, and LUS at *T*_0_ and *T*_1_, and reported it as days of life (DoL) and post-menstrual age (PMA). Results are summarized in Table 2.

**Table 2:** Time for achieving the main nutrition milestones (i.e., full enteral feeding, start of bottle feeding, total oral feeding) related GA at birth, and LUS at *T*_0_.

Milestone	Predictive factor	*r*	RC	CI95%	*p*-value
Full enteral feeding (PMA)	Constant		20.54	12.03–29.05	<0.001
	GA (weeks)	0.617	0.40	0.11–0.70	0.011
Full enteral feeding (day)	Constant		159.13	92.79–225.46	<0.001
	GA (weeks)	−0.770	−4.84	−7.14 (−) −2.55	<0.001
1st meal by bottle (day)	Constant		240.60	178.54–302.68	<0.001
	GA (weeks)	−0.832	−8.01	−10.04 (−) −5.98	<0.001
	*T*_0_ LUS score	0.343	1.96	0.75–3.16	0.004
1st meal by bottle (PMA)	Constant		31.70	30.30–33.10	<0.001
	*T*_0_ LUS score	0.676	0.20	0.07–0.33	0.006
Total oral feeding (day)	Constant		217.00	184.08–357.92	<0.001
	GA (weeks)	−0.897	−8	−10.97 (−) −5.03	<0.001

r, Pearson correlation coefficient; RC, regression coefficient; CI, confidence interval.

The achievement of full enteral feeding correlates with GA at birth (*p* < 0.001) but not with LUS scoring (*p* n.s.).

The introduction of the first meal by bottle correlates both with gestational age (*p* < 0.001) and ultrasound scores (*p* = 0.004). In particular, indeed, the day of life on which the first meal is introduced depends on GA at birth and on *T*_0_ ultrasound score (*p* < 0.001), while PMA at the time of this nutritional milestone is influenced by the *T*_1_ LUS (*p* = 0.006), while not by GA at birth (*p* n.s.).

In order to investigate factors likely predictive of total days of hospitalization, we firstly performed a univariate analysis, then a step-wise multivariate analysis (Table 3). We included in the multivariate analysis: *T*_0_ LUS score, *T*_5_ LUS score, *T*_1_ LUS score, the timing of each nutritional milestone, the duration of oxygen supplementation, and diagnosis of bronchopulmonary dysplasia. The other clinical variables were not included, due to their low prevalence in our study population. Only the length of need for oxygen supply was significant (*p* = 0.003), while the timing of full oral nutrition showed a trend to significance (*p* = 0.06). In the multivariate analysis, only this latter factor reached a statistical significance (*p* = 0.005).

**Table 3:** Evaluation of predictive factors towards days of hospitalization.

Correlation with days of hospitalization			
Univariate analysis			Step-wise MLR
Investigated factors			
GA (weeks)	Pearson	−0.649	
	Sign. (two tails)	0.016	0.085
	*N*	13	
1st meal by bottle (day)	Pearson	0.776	
	Sign. (two tails)	0.002	
	*N*	13	
Full enteral feeding (day)	Pearson	0.582	
	Sign. (two tails)	0.037	
	*N*	13	
Total enteral feeding (day)	Pearson	0.794	
	Sign. (two tails)	0.004	0.062
	*N*	11	
*T*_0_ LUS score	Pearson	0.279	
	Sign. (two tails)	0.356	
	*N*	13	
BPD	Pearson	0.694	
	Sign. (two tails)	0.008	
	*N*	13	
Oxygen dependence (days)	Pearson	0.789	0.805
	Sign. (two tails)	0.001	0.003
	*N*	13	
*T*_1_ LUS score	Pearson	0.621	
	Sign. (two tails)	0.023	
	*N*	13	
*T*_5_ LUS score	Pearson	0.679	
	Sign. (two tails)	0.011	
	*N*	13	

GA, gestational age; BPD, bronchopulmonary dysplasia.

Finally, we searched for a possible correlation between LUS and respiratory outcomes. *T*_1_ LUS score resulted predictive for length of invasive/non-invasive respiratory support (*p* = 0.002) and length of oxygen supply (*p* = 0.001), while *T*_0_ LUS score did not (*p* n.s.).

Anecdotally, we report here that 2 out of 3 neonates excluded from statistical analysis because of suffering from genetic syndrome (i.e., Silver-Russell Syndrome and Rubistein-Taybi Syndrome) presented with meal-related symptoms (desaturation, bradycardia, regurgitation). In both of them we performed LUS, but only for the second one, who had consolidations showed by the ultrasounds, VFSS confirmed episodes of reflux and stagnation of the contrast agent in the nasopharynx, poor pharyngeal coordination, and multiple episodes of inhalation.

## 4. Discussion

LUS demonstrated feasibility and reliability in many clinical contexts, including pediatric and adults settings, acute as well as chronic primary and secondary pulmonary conditions. A recent paper reported that operators with limited experience in the neonatal lung ultrasound field (neonatologists undergoing a 2-hours theorical training, 30 min of familiarization with the probe and a practical part supervised by an expert operator including 25 studies) obtained evaluations comparable to those of expert operators. Such a rapid learning curve encourages the approach to the method and is a relevant point in favor of its diffusion and generalizability (20, 21). The main contributors to this aspect are the limited complexity of the technique *per se* compared to other ultrasound methods and the precise iconography of the findings, with a greater immediacy in understanding the images in most clinical conditions where its utility and diagnostic accuracy has been demonstrated. These aspects encourage potential applications of the LUS technique for non-invasive, infant-friendly study in fragile populations such as newborns and infants. These has been recently addressed by our group supporting the usefulness of LUS to identify pulmonary abnormalities potentially related to feeding difficulties in infants and young children with neurological impairment (8, 9). In this direction, infants born preterm or newborns with other comorbidities that may impact feeding abilities may represent a potential target to promote a further use of LUS. There are however a number of theoretical issues that contribute to the variety of LUS findings from the earliest stages of life, that rapidly vary since adaptation to the extra uterine environment. Therefore, direct observation of LUS at earlier stages and in target population such as preterm newborns can contribute to the understanding LUS feasibility and clinical implication in relation with feeding skills development.

Our findings support the feasibility of the use of this technique in a small sample of preterm newborns. However, group analyses in our sample of subjects by applying a previously described LUS abnormalities scoring system showed no differences between pre and post meal LUS acquisition.

Differently than reported in infants and toddlers (8), in our neonatal population LUS before and after meal did not show significantly different results. We interpreted this result considering a few aspects. First of all, we have to underline that our study was limited by a small number of enrolled cases since it was a pilot study.

As forwards the tool itself, we have to consider the ultrasound score linked to pulmonary conditions. Indeed, preterm infants who still need intensive or sub-intensive care when compared to older outpatient children with neurological impairment, have a greater probability of pulmonary involvement, presenting LUS B-lines, so with a pathologic score. The presence of multiple B-lines (in our study if numbered ≥5) was defined by score 2, but encompasses a continuum that reaches up to the white lung. Numerical variations of the B lines in this spectrum do not cause scoring variations, with potentially significant loss of information.

A possible explanation for our findings related to the fact that systematic evaluation of pulmonary changes following symptomatic regurgitation was not included, which might be hypothesized to be an event related to ultrasound signs suggestive of retrograde inhalation. Indeed, regurgitation can occur with a variable latency from the end of the meal, and it is difficult to predict: this means that the operator is not always available/present in the ward to carry out an *ad hoc* study. Furthermore, even when this coincidence occurs, it is still a post-event ultrasound evaluation, therefore difficult to interpret. Even more difficult to classify are the silent retrograde inhalations, for which it is possible to hypothesize only the evaluation of the cumulative impact of recurring events over time. VFSS can identify retrograde barium meal inhalations only if these occur shortly after food intake, given the short duration of the exam, while FEES can possibly highlight indirect signs of gastroesophageal reflux disease. In conclusion, the negativity of these analyses does not exclude the possibility that food material enters the airways.

Despite our group analysis was not sensitive in meal related lung abnormalities, some considerations about single cases may be conducted. In particular, the anecdotic report about neonates with genetic syndromes suggests a possible role of pulmonary consolidation detections, in the diagnostic suspicion of inhalation when symptoms meal-related occur, although those patients were excluded from the statistical data analysis.

To evaluate the correlation of age-related ultrasound evaluations with the timing of achievement of the different nutritional milestones, a multivariate analysis was conducted considering GA at birth. In fact, this is a factor recognized in the literature as a predictor of the development of eating skills (22). Our study showed that only GA correlates with the timing of achievement of full enteral feeding: this data can be interpreted on the basis of the fact that the rate of increase of enteral feeding is more influenced by the immaturity of digestive functions (inversely related to GA) than by the respiratory condition *per se*, when orogastric tube feeding is used as well as in our population.

On the contrary, feeding by bottle seems to correlate both with GA at birth and with LUS scores. In particular, the day of life on which the first bottle is introduced depends on GA at birth and on *T*_0_ LUS score, while PMA at the time of this stage correlates with the *T*_1_ LUS. The LUS predictivity could depend on the worse performance during swallowing apnea, with consequent less control of the parameters during the meal, given by a more severe pulmonary condition. The results obtained by other Authors (23, 24) regarding the maturation of alimentary skills of infants with bronchopulmonary dysplasia (BPD) support our speculation. In the sample analyzed by Park, patients with BPD start enteral feeding and reach complete enteral feeding at a PMA comparable to non-BPD patients, from whom they differ significantly in terms of the timing of acquisition of oral nutritional milestones.

In our study cohort, PMA at the introduction of the first bottle meal correlates with *T*_1_ LUS score, canceling out the statistical contribution given by GA at birth. The data is particularly interesting in comparison with what reported by Amaizu and coll (25). about the influence of GA on the timing for the acquisition of successful oral feeding. Indeed, it is confirmed that there is no close relationship between GA and PMA at the start of oral feeding, and a new possible predictive element is introduced, such as lung ultrasound findings. Patterns of lung abnormalities might reflect direct or indirect pulmonary status related to successful acquisition of oral feeding. In our population of preterm infants, the timing for the achievement of total oral feeding depends only on GA at birth, while none of the investigated parameters seems to be predictive of PMA upon reaching this stage. Further perspective studies are mandatory to support this hypothesis.

Previous data about the predictive factors of preterm infants hospitalization are inconclusive, underlining the complexity of these patients as the real main predictive factor, and paying particular attention to the presence of late complications rather than perinatal conditions (26). Our statistical analysis concluded that only oxygen supply duration and the time necessary to achieve total enteral feeding were predictive of the time for discharge. Therefore, our data agree with previous reports, since oxygen dependence is a major pointer of patient's general health status. Safe total oral feeding represents an important stage in the evaluation for a patient's discharge, and highlights how also the development of feeding skills can reflect and summarize the global complexity of a newborn, as already reported by Edwards and collaborators (27).

The predictivity of *T*_1_ LUS score towards the days of total respiratory assistance and the days of oxygen-dependence represents an important starting point for reflection, in our opinion. The ever-increasing attention to the prevention of complications rather than to their treatment, involves the search for early risk indicators and the clinical management individualization, as also underlined by the study by Rodriguez-Fanjul and collaborators (28) concerning the administration of surfactant guided by ultrasound criteria rather than by ventilator criteria, or by that of the Alonso-Ojembarrena group (29, 30) on the predictivity of the ultrasound score in the diagnosis of bronchodysplasia.

The predictive role of the early ultrasound score, which in our sample prevails over that given by GA at birth, could represent the starting point for tailoring care of the newborn, by setting up dedicated and early therapeutic and care protocols. A combined intervention seems to likely have a beneficial effect also on oral feeding proficiency in preterm infants.

In conclusion, our study did not allow us to attribute a certain value to the ultrasound monitoring of the meal in high-grade preterm infants, maybe due to the widely discussed limits, mainly related to the sample size and the critical clinical situations. However, clinical observation has allowed us to focus attention on a possible suspicious ultrasound pattern, such as that characterized by the appearance of consolidations with a chronic-relapsing trend. The description is currently anecdotal, due to the small number of patients recruited and the lack of a systematic radiographic evaluation of reflux in all patients with suspected retrograde inhalation, but could represent the basis for future correlation studies. Further prospective studies with medium-long term outcome of feeding and global development including LUS findings, will allow a better interpretation and validation of these preliminarily findings.

LUS is useful in diagnosis and management of neonatal respiratory distress. Respiratory diseases influence feeding milestones. In this sense, LUS can be an indirect tool to preview feeding abilities. Anyway, as statistical analysis shows, in our cohort there was not a direct predictive capacity. Therefore, we suppose that LUS can be an indirect tool to preview feeding abilities.

LUS monitoring of the nutritional development stages, not yet proposed in the neonatal field to the best of our knowledge, has also made it possible to identify a possible investigation focus for future studies aimed to tailor strategies and timing for oral feeding in preterm infants. Nevertheless, in neonates, especially preterm infants, LUS scoring might be optimized to discern features specifically due to silent inhalations from respiratory distress patterns.

Moreover, LUS study may also represent an important extension of the respiratory evaluation of the preterm, a chapter yet to be written and which can contribute significantly to the management of respiratory tools, supportive therapies and the understanding of the mechanisms underlying chronic complications such as BPD. Its extensive use is made possible by the minimal biological impact on the patient, by its low invasiveness and the consequent safety of use even on the unstable patient, by the portability of the instrumentation and by the rapid initial learning curve. However, our results underline the importance of an extensive and continuous practice of lung ultrasound on the preterm infant, that can only be partially included in the current scoring methodologies, due to its clinical complexity.

## Data Availability

The raw data supporting the conclusions of this article will be made available by the authors, without undue reservation.
